# Investigation of Advertising and Food Environment Surrounding Primary Schools in New Zealand

**DOI:** 10.1002/hpja.70097

**Published:** 2025-09-14

**Authors:** Kate Nicholls, Ajmol Ali, Carol Wham

**Affiliations:** ^1^ School of Sport, Exercise and Nutrition Massey University Auckland New Zealand

**Keywords:** child obesity, food and beverage advertising, Google street view, health, unhealthy

## Abstract

**Objective:**

The objective of this study is to examine food and beverage advertising and stores around New Zealand (NZ) primary schools.

**Methods:**

Food and beverage advertising and stores within 800 m of 18 primary schools in NZ were collected in August 2023. Food and beverage advertising and store characteristics were compared across low (1–3), medium (4–7) and high (8–10) decile schools, reflecting socioeconomic status.

**Results:**

‘Non‐core’ food and beverage advertisements (*n* = 426, 89%) outnumbered ‘core’ (*n* = 53, 11%) with sugar‐sweetened beverages (*n* = 192, 40.1%) the most common category, and Coca‐Cola the most common brand (*n* = 158, 33%, *p* = 0.033). Food stores were the most common location for food and beverage advertisements (97.1%, *p* ≤ 0.001). More food and beverage advertisements surrounded low (*n* = 406, 43%) and medium (*n* = 208, 43.4%) decile schools, with low‐decile schools having more nearby advertisements (0–400 m) (*n* = 111, 57.5%). The most common food and beverage stores near schools were local fast‐food (44.7%) and convenience stores (24.2%). Store type varied by school decile, with more convenience stores (51.9%) and fast‐food chains (81.3%) near low‐decile schools.

**Conclusions:**

‘Non‐core’ food and beverage advertising, local fast‐food, fast‐food chains and convenience stores were prevalent surrounding primary schools, particularly low and medium‐decile schools.

**So What?:**

These findings highlight high levels of exposure to unhealthy food and beverage advertising and food stores, especially surrounding schools within low and medium socioeconomic areas.

## 1 Introduction and Objective

Childhood obesity is a prevalent global health concern [1]. In NZ, the impact is profound, affecting 1 in 10 children [2]. The World Health Organization (WHO) has outlined a comprehensive action plan featuring six objectives to address this issue, one of which emphasises the creation of health‐promoting environments as a means to mitigate modifiable risk factors associated with obesity [3].

Food environments are a complex interplay of physical, economic, policy and sociocultural factors that influence individuals' dietary patterns and health [4]. Specifically, food environment refers to both the foods and beverages advertised (outdoors and on a stationary object) surrounding schools and the food and beverage stores including convenience stores, bakeries, fast‐food chains, local fast‐food, cafés and supermarkets/produce stores surrounding schools. Exposure to unhealthy food and beverage environments, including advertising [5, 6, 7, 8] as well as stores that sell unhealthy products [9], can have a detrimental impact on children's dietary patterns and health.

The NZ food environment in which children live is unhealthy, mainly constituting a high volume of both unhealthy food and beverage advertisements and stores [10, 11, 12, 13, 14, 15, 16, 17, 18, 19, 20] and is accentuated in lower socioeconomic neighbourhoods [10, 12, 13, 15, 18, 19].

New Zealand authorities have taken steps to promote healthier environments through initiatives like Healthy Active Learning, launched in 2020. This government‐funded initiative, involving Sport NZ, Health NZ/Ministry of Health and Ministry of Education, aims to improve children's health by enhancing food, drink and physical activity environments in schools [21]. As outlined in the Healthy Food and Drink Guidance for Schools, school menus are analysed using a ‘traffic light’ system: ‘green’ for most nutritious, ‘amber’ for moderately nutritious and ‘red’ for least nutritious items [22]. Schools can also adopt policies to increase ‘green’ foods and restrict ‘red’ options, such as the *Healthy Food and Drink Policy* as well as a *Water and Milk only Policy*. However, evidence suggests that interventions solely within schools may be insufficient; the broader food environment around schools also needs consideration [23]. Nearly two‐fifths of children (38.8%) aged 5–14 years use active transport (like cycling and walking) to travel to and from school [24], which means they have opportunities to be exposed to advertising and promotion en route.

In NZ, the Children and Young People's Advertising Code established in 2017 aims to regulate advertisements for occasional food and beverage products targeting children, especially in areas where children gather, such as schools [25]. However, the code's vagueness and inadequate regulation have prevented its effectiveness [26]. In 2024, a new code was introduced to enhance the protection of children from targeted advertising. Despite this update, the new code, like its predecessor, continues to face challenges due to inadequate regulation and enforcement. To address some of these concerns, the Out of Home Media Association Aotearoa (OOHMAA) introduced a Placement Policy in 2020, aimed at limiting children's exposure to advertisements for occasional food and beverage products [27]. Whilst OOHMAA's initiative reflects a proactive approach by the industry, it remains a self‐regulatory measure, raising similar concerns about its effectiveness without legal enforcement. The WHO has recommended that countries implement mandatory policies to protect children from unhealthy food and beverage advertising ([28]) and several countries worldwide, including South Korea, the United Kingdom, Ireland, the United States and the Philippines, have enacted zoning measures to regulate such advertisements near schools. However, NZ has yet to adopt these stricter measures.

Currently, NZ research that has examined the food environment, encompassing advertisements surrounding primary schools, has focused on specific regions, such as Auckland [10, 14, 15] or Wellington [16, 17, 20]. Studies have examined advertisements in one location, such as at bus stops [15] or convenience stores [10] and in one case outdoor advertisements surrounding primary schools in NZ [18]. Similarly, only a few studies have investigated the type and number of food stores surrounding primary schools in NZ [11, 12, 13, 16, 19, 20], of which, only two examined stores across NZ schools [19, 29].

One NZ study found the concentration of food and beverage advertisements and outlets significantly affected efforts to improve the school food environment [20]. The present study aims to investigate the external food environment (food and beverage advertisements) and presence of food stores surrounding a convenience sample of primary schools across NZ. We will also explore whether there is any relationship between the healthiness of school food menus (‘green,’ ‘amber’ and ‘red’ menu items) and food advertisements and whether the number of food advertisements around schools has any relationship to the presence of school food policies.

## 2 Methodology

### 2.1 Study Design

This was an observational cross‐sectional study involving 18 primary schools across NZ. Utilising Google Street View, a distance of 800 m surrounding each school was travelled from the main entrance of each school, gathering data on surrounding stores and food and beverage advertisements. Ethics approval was provided by Massey University (Low‐Risk approval 4000027336).

### 2.2 School Selection

All primary schools (contributing [Years 1–6], full primary [Years 1–8] and intermediate [Years 7–8]) involved in the Healthy Active Learning initiative were invited to complete the food policy and practices questionnaires and provide sample menus. Data were collected from 18 primary schools where food menu and policy data acquired from the Healthy Active Learning initiative overlapped. The characteristics of each school, compared to all NZ primary schools [30], are shown in Table 1.

**TABLE 1:** School characteristics.

Characteristic	Sample, *n* (%)	All NZ primary schools, *n* (%)
Total	18 (100)	1825 (100)
Type of school		
Contributing (years 1–6; typically ages 5–11)	12 (66.6)	768 (42.1)
Full primary (years 1–8; typically ages 5–13)	6 (33.3)	1057 (57.9)
Region		
Northland	2 (11.1)	105 (5.8)
Auckland	9 (50.0)	369 (20.2)
Bay of plenty	3 (16.7)	112 (6.1)
Wellington	2 (11.1)	175 (9.6)
Otago/Southland	2 (11.1)	178 (9.8)
Urban/rural		
Urban	13 (72.2)	1222 (67.0)
Rural	5 (27.8)	603 (33.0)
Decile		
Low (1–3)	4 (22.2)	—
Medium (4–7)	7 (38.9)	—
High (8–10)	7 (38.9)	—

In NZ, until 2023, schools used the decile score system to assess the socioeconomic context of the student population. This score influenced funding allocation and resource distribution to address educational disparities; decile 1 schools had the highest proportion whilst decile 10 schools had the lowest proportion of students from low socioeconomic communities [31].

Deciles were grouped into three categories: low (1–3), medium (4–7) and high (8–10), aligning with similar studies in NZ [10, 14, 15].

### 2.3 Google Street View

Google Street View's omnidirectional imagery was utilised for data collection, chosen for its ethical attributes [32] and efficiency in gathering information on outdoor food environments [14, 15]. The most recent images uploaded to GSV were used, between August 2019 and May 2023.

### 2.4 Data Collection – Advertisements

Eligibility for advertisements was determined by referencing an international collaboration of researchers and health organisations called INFORMAS (International Network for Food and Obesity/Non‐communicable Diseases Research, Monitoring, and Action Support) protocol [33] as well as a protocol developed to capture advertisements near schools [34]. Advertisements were eligible for data collection if they: (a) contained a food or non‐alcoholic beverage, (b) were within 800 m walking distance from the school studied, (c) were branded, and (d) were outdoors on a stationary object. Advertisements that featured a food/non‐alcoholic beverage brand without referring specifically to the product were included. However, advertisements that primarily served as store identification were excluded.

Data collected for each eligible advertisement included: (a) distance from the school, (b) setting of the advertisement, (c) food and beverage store category, (d) type of the advertisement, (e) brand name, (f) food/beverage category (‘core’/’non‐core’), (g) food/beverage category (green, amber, red), (h) primary marketing classification, (i) secondary marketing classifications and (j) GSV date.

**FIGURE 1:**
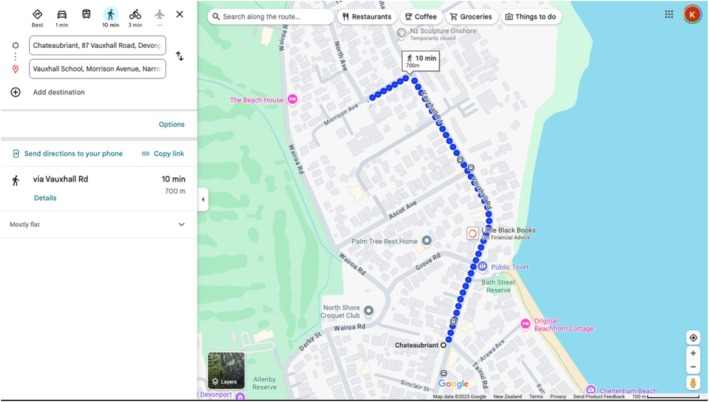
Example screenshot of to show ‘virtual’ walking distance from main entrance of school to a food store/advertising.

A walking distance of 800 m from each school was investigated, as this is considered a walkable distance for primary school‐aged children, equating to approximately 10 min [11, 14]. Using the school entrance specified by Google Maps [34], each street was travelled in both directions, up to 800 m, to avoid missing any advertisements [15]. To determine the distance of the advertisement from the school's main entrance, the walking distance function on Google Maps provided a distance between the two points (Figure 1). The collected data, being categorical, was grouped into 0–400 m or 401–800 m to assess the proximity of advertisements to the school.

The outdoor food and beverage advertisement collection protocol developed by INFORMAS focused on monitoring food environments related to obesity and non‐communicable diseases. This protocol, used for data collection, includes details on each advertisement's setting, type and brand [33]. A specific data collection protocol designed for capturing advertisements near schools [34] guided the categorisation of advertisements into ‘core’ and ‘non‐core’ food and beverage categories and defined their primary marketing classifications.

Food and beverage advertisements were categorised into ‘core’ and ‘non‐core’ terminology using the WHO nutrient profiling system ([28]), a model specifically created to limit the marketing of foods to children. Additionally, these advertisements were categorised according to the Healthy Food and Drink Guidance traffic light system [22], enabling a comparison between the proportions of ‘green’, ‘amber’ and ‘red’ advertisements near schools and the corresponding proportions of ‘green’, ‘amber’ and ‘red’ food menu items available within the schools. In 2022, the national leadership for the health component of Healthy Active Learning transitioned to Health NZ's National Public Health Service, and Health NZ is referenced throughout this paper for consistency with current governance.

Primary marketing classifications specified the use of promotional characters by their target audience as either ‘child only’ or ‘child and adult’. Secondary marketing classifications refer to other specific niche‐marketing techniques that may be seen, including promotion/discount, contest, limited/special edition, gift or collectable, loyalty programmes, supporting charities, app marketing and exaggerated portion sizes. These secondary marketing categories were adapted from established protocols [33, 34]. More detailed methods can be found in Egli et al. [7] and Mackay et al. [33].

Food and beverage store category was recorded for all advertisements within the property boundaries of a food store, including on the footpath in front of the food store. The categories include convenience stores, bakeries, fast‐food chains, local fast‐food, café, supermarkets/produce stores and others. Convenience stores included dairies, superettes, mini‐marts and food marts. Bakeries were local or a chain (e.g., Baker's Delight). A fast‐food chain was any food store within a chain, such as KFC, McDonald's or Subway. Local fast‐food included any food store not part of a chain or franchise, including local restaurants and food trucks. Supermarkets included all large stores such as Pak'nSave and New World, fruit and vegetable stores, and other produce stores.

### 2.5 Data Collection – Stores

Food and beverage stores were eligible for data collection if they: (a) were within 800 m walking distance of the school selected and (b) sold primarily food or non‐alcoholic beverages. The following was collected for each food and beverage store: (a) distance from the school, (b) store category, and (c) Google Street View date.

### 2.6 Analysis of Healthy Active Learning Data

The food menus and policies baseline data were collected from primary schools across NZ as part of the evaluation of the Healthy Active Learning initiative conducted by the Massey University research team between November 2020 and March 2021.

The school menus assessed within the Healthy Active Learning initiative utilised a food classification system established by the Ministry of Health [22]. This system employs a traffic light model which allows for comparison of different menu items as either ‘green’ (most nutritious foods), ‘amber’ (moderately nutritious) or ‘red’ (least nutritious). The proportions of each of these foods on the school menus were calculated.

The same classification system was applied to food and beverage advertisements around each school, determining the overall proportions of ‘green’, ‘amber’ and ‘red’ advertisements. The proportions of ‘green’, ‘amber’ and ‘red’ advertisements were compared with the proportions of menu items to identify potential correlations.

Food policy data collected from Healthy Active Learning was also utilised, including the presence of a *Healthy Food and Drink Policy* and the *Water and Milk only Policy* within each school. The *Healthy Food and Drink Policy* aimed to increase the availability of healthy food options within environments such as primary schools and uses the traffic light system, whereby ≥ 55% of all products on offer must fit into the ‘green’ category, as well as providing other guidelines on suitable foods [22]. The water and milk only policy was designed specifically for schools to make water and plain milk the only drinks available in schools. The analysis aimed to explore relationships between the healthiness of school environments and the advertising surroundings by comparing the presence of these policies with the number of advertisements around each school.

### 2.7 Statistical Analysis

Data were downloaded from Microsoft Excel and analysed using IBM SPSS statistical package version 29 (IBM Corporation, New York, USA). Descriptive statistics assessed the number of advertisements and stores surrounding schools. Chi‐square testing and post hoc testing determined whether school decile was associated with advertisement characteristics. Cramer's *V* assessed the strength of the association, categorised as weak for 0–0.3, moderate ≥ 0.3 to < 0.5, and strong ≥ 0.5 to 1 [35]. Categories were condensed to adhere to Chi‐square test assumptions. ‘Core’ food and beverage advertisements were combined, as were ‘non‐core’ food and beverage advertisements. Primary marketing classifications were separated into ‘child only’ and ‘child and adult’ categories. Secondary marketing classifications were categorised as ‘none present’ and ‘present’. Fisher's exact testing determined the association between decile and food and beverage store categories as Chi‐square test assumptions were unmet.

Spearman's rank correlation analysis was used to investigate the relationships between ‘green’, ‘amber’ and ‘red’ menu items and advertisements. Furthermore, correlations were undertaken between the number of advertisements around schools and the presence of school policies, including healthy food and drinks or water and milk only. Statistical significance was determined with a threshold of *p* < 0.05.

## 3 Results

### 3.1 Characteristics of Schools

The 18 primary schools investigated were from five regions across NZ, covering both the North and South Islands, urban and rural areas. Auckland primary schools comprised 20.2% of the total number of primary schools, equalling 50% of the sample population, resulting in slight over‐representation. Equal numbers of schools from each decile grouping were investigated; therefore, there was a fair representation of NZ's overall population regarding socioeconomic status.

### 3.2 Characteristics of Advertisements

A total of 479 food and beverage advertisements were collected (Table 2). The majority were found near low (*n* = 406, 43%) and medium (*n* = 208, 43.4%) decile schools, particularly in Auckland (*n* = 375, 78.3%). More advertisements were located beyond 400 m (*n* = 286, 59.7%) compared to within 400 m (*n* = 193, 40.3%). Most were on food and beverage stores (*n* = 465, 97.1%), and primarily convenience stores (*n* = 361, 80.4%). Signage and free‐standing signs were the most common types. In total, 45 different brands were identified, with Coca‐Cola as the most prevalent (*n* = 158, 33%), followed by Tip‐top, Monster, Anchor and Streets. The top categories were beverage ‘non‐core’: sugar‐sweetened (*n* = 192, 40.1%) and ‘non‐core’ foods (*n* = 152, 31.7%). Few were marketed to children with cartoon (*n* = 13, 2.7%) or licenced characters (*n* = 2, 0.4%), whilst the majority targeted both children and adults. The most common type of secondary marketing seen was promotion/discounts (*n* = 33, 6.9%).

**TABLE 2:** Characteristics of food and beverage advertisements.

Characteristic	Subcategory	Number of advertisements, *n* (%)
School decile	Low (1–3)	206 (43.0)
	Medium (4–7)	208 (43.4)
	High (8–10)	65 (13.6)
Region	Northland	2 (0.4)
	Auckland	375 (78.3)
	Bay of Plenty	67 (14.0)
	Wellington	27 (5.6)
	Otago/Southland	8 (1.7)
Walking distance from school (m)	0–400	193 (40.3)
	401–800	286 (59.7)
Setting	Food and beverage store	465 (97.1)
	Road/footpath	0 (0)
	Building	8 (1.7)
	Bus shelter	2 (0.4)
	Train station/stop	4 (0.8)
	Mobile cart/stall or vending machine	0 (0.0)
Food and beverage store category^a^	Convenience	361 (80.4)
	Bakery	14 (3.1)
	Fast‐food chain	33 (7.3)
	Local fast‐food	36 (8.1)
	Cafe	0 (0.0)
	Supermarket	5 (1.1)
	Other	0 (0)
Type	Billboard	0 (0)
	Sign/poster/banner	340 (80.0)
	Free‐standing sign	138 (28.8)
	Painted building/wall	1 (0.2)
	Digital signs/LED	0 (0)
	Other	0 (0)
Food/beverage brand name^b^	Coca‐Cola	158 (33.0)
	Tip‐top	58 (12.0)
	Monster	34 (7.0)
	Anchor	32 (7.0)
	Streets	29 (6.0)
	Other	168 (35.0)
Food/beverage category (‘core’/‘non‐core’)	Food ‘non‐core’	152 (31.7)
	Food ‘core’	16 (3.3)
	Beverage ‘non‐core’: sugar‐sweetened	192 (40.1)
	Beverage ‘non‐core’: energy drink	77 (16.1)
	Beverage ‘non‐core’: coffee	5 (1.0)
	Beverage ‘core’	37 (7.7)
	Other	0 (0)
Food/beverage category (green, amber or red)^c^	Green	56 (11.7)
	Amber	13 (2.7)
	Red	410 (85.6)
Primary marketing classification	Child only: company owned cartoon character	13 (2.7)
	Child only: licenced TV or movie character	2 (0.4)
	Child and adult: brand/product	431 (90.0)
	Child and adult: random person	28 (5.8)
	Child and adult: famous non‐sports people/influencers/celebrity	3 (0.7)
	Child and adult: amateur sportsperson/team	1 (0.2)
	Child and adult: famous sportsperson/team	1 (0.2)
Secondary marketing classification	No premium/portion distortion present	430 (89.8)
	Promotion/discount	33 (6.9)
	Contest	5 (1.0)
	Limited/special edition	0 (0)
	Gift or collectable	0 (0)
	Loyalty programme	1 (0.2)
	Supporting charity	0 (0)
	App marketing	2 (0.4)
	Exaggerated portion size	8 (1.7)

^a^ This includes only advertisements associated with a food and beverage store, excluding those not associated (*n* = 30, 6.3%).

^b^ Includes top five brands found, all other brands listed as ‘other’.

^c^ Based on the Healthy Food and Drink Guidance in schools, advertisements are classified into colour codes of green, amber and red [22].

### 3.3 Characteristics of Stores

A total of 215 food and beverage stores were identified (Table 3). The majority were situated near low (*n* = 86, 40.2%) and medium (*n* = 85, 39.5%) decile schools, with fewer near high‐decile schools (*n* = 44, 20.5%). More food and beverage stores were located further away from schools, between 401 and 800 m (*n* = 127, 59.1%), as opposed to 0–400 m from schools (*n* = 88, 40.9%). Local fast‐food outlets were the most prevalent (*n* = 96, 44.7%), followed by convenience stores (*n* = 52, 24.2%).

**TABLE 3:** Characteristics of food and beverage stores.

Characteristic	Subcategory	Number of food and beverage stores, *n* (%)
School decile	Low (1–3)	86 (40.0)
	Medium (4–7)	85 (39.5)
	High (8–10)	44 (20.5)
Region	Northland	1 (0.5)
	Auckland	185 (86.0)
	Bay of Plenty	18 (8.4)
	Wellington	8 (3.7)
	Otago/Southland	3 (1.4)
Walking distance from school (m)	0–400	88 (40.9)
	401–800	127 (59.1)
Food and beverage store category	Convenience	52 (24.2)
	Bakery	20 (9.3)
	Fast‐food chain	16 (7.4)
	Local fast‐food	96 (44.7)
	Cafe	22 (10.2)
	Supermarket	8 (3.7)
	Other	1 (0.5)

### 3.4 Correlation Between Advertisements and Stores

There was a strong positive correlation between the number of food and beverage advertisements and stores surrounding schools (*r*_s_ = 0.879, *n* = 18, *p* < 0.001). Therefore, subsequent data analysis will focus exclusively on advertisements.

### 3.5 Decile Versus Advertisement Characteristics

Various associations were identified between school decile level and advertisement characteristics (Table 4). School decile demonstrated a moderate association with the proximity of advertisements (*X* ^2^(2) = 47.856, *p* < 0.001, *V* = 0.316). For the 0–400 m subcategory, low (*n* = 111, 57.5%) and medium‐decile schools (*n* = 47, 24.4%) exhibited a statistically significant difference compared to high‐decile schools (*n* = 35, 18.1%), indicating that low and medium‐decile schools had more advertisements in closer proximity. For the 400–800 m category, low (*n* = 95, 33.2%) and medium‐decile schools (*n* = 161, 56.3%) also had more advertisements than high‐decile schools (*n* = 30, 10.5%), reinforcing the trend that lower‐decile schools are associated with a higher number of advertisements.

**TABLE 4:** Decile versus advertisement characteristics.

Advertisement characteristic	Subcategory	Decile			Total, *n* (%)	*p*	Effect size (interpretation)
		Low, *n* (%)	Medium, *n* (%)	High, *n* (%)			
Distance from school (m)	0–400	111 (57.5)_a_	47 (24.4)_a_	35 (18.1)_b_	193 (100)	< 0.001	0.316 (moderate)
	401–800	95 (33.2)_a_	161 (56.3)_a_	30 (10.5)_b_	286 (100)		
Food and beverage category	‘Non‐core’ food and beverages	184 (42.7)_a_	189 (43.9)_a_	58 (13.5)_a_	431 (100)	0.852	**—**
	‘Core’ food and beverages	22 (45.8)_a_	19 (39.6)_a_	7 (14.6)_a_	48 (100)		
Brand^a^	Coca‐Cola	81 (51.3)_a_	57 (36.1)_b_	20 (12.7)_a,b_	158 (100)	0.033	0.119 (weak)
	Other	125 (38.9)_a_	151 (47.0)_b_	45 (14.0)_a,b_	321 (100)		
Primary marketing classification	‘Child only’	7 (46.7)_a_	4 (26.7)_a_	4 (26.7)_a_	15 (100)	0.222	**—**
	‘Child and adult’	199 (42.9)_a_	204 (44.0)_a_	61 (13.1)_a_	464 (100)		
Secondary marketing classification	Present	19 (38.8)_a,b_	29 (59.2)_b_	1 (2.0)_a_	49 (100)	0.013	0.135 (weak)
	None present	187 (43.5)_a,b_	179 (41.6)_b_	64 (14.9)_a_	430 (100)		

*Note:* Statistical analysis conducted using a Chi‐square test with post hoc Cramer's *V* showing the strength of association. Subscript letters (a and b) indicate the presence of statistical significance between each decile in each subcategory. Categories assigned different subscript letters are statistically significant (*p* < 0.05).

^a^ Only the brand Coca‐Cola is displayed, as it was the most common brand.

For Coca‐Cola advertisements, a weak association with school decile was observed (*X* ^2^(2) = 6.816, *p* = 0.033, *V* = 0.119). Differences were found between low‐decile (*n* = 81, 51.3%) and medium‐decile (*n* = 57, 36.1%) schools, suggesting that low‐decile schools had a greater proportion of Coca‐Cola advertisements compared to medium‐decile schools. However, no significant difference existed between the number of Coca‐Cola advertisements surrounding high‐decile schools compared to low and medium‐decile schools, indicating that high‐decile schools do not experience a similar level of exposure to these advertisements.

There was a weak association between the presence of secondary marketing classifications of advertisements and school decile (*X* ^2^(2) = 8.696, *p* = 0.013, *V* = 0.135). Medium‐decile schools had a higher proportion of advertisements both with (*n* = 29, 59.2%) and without (*n* = 179, 41.6%) secondary marketing classifications than high‐decile schools (*n* = 1, 2.0% and *n* = 64, 14.9%).

No significant associations were found between school decile and food/beverage category (*p* = 0.852) or primary marketing classifications (*p* = 0.222).

### 3.6 Decile Versus Store Characteristics

School decile was associated with food and beverage store characteristics (*p* < 0.001; Table 5). Convenience stores, bakeries, local fast‐food and fast‐food chains were more prevalent around low (51.9%, 40%, 32.3%, 81.3%) and medium‐decile schools (20.6%, 50%, 43.8%, 18.8%) compared to high‐decile schools (5.8%, 10%, 24%, 0.0%). Conversely, high‐decile schools had a higher proportion of supermarkets (37.5%) and cafés (59.1%) than low‐decile schools (12.5%, 22.7%).

**TABLE 5:** Decile versus food and beverage store characteristics.

Store characteristic	Subcategory	Decile			Total, *n* (%)	*p*
		Low, *n* (%)	Medium, *n* (%)	High, *n* (%)		
Food and beverage store category	Convenience	27 (51.9%)	22 (20.6)	3 (5.8%)	52 (100)	< 0.001
	Bakery	8 (40.0%)	10 (50.0%)	2 (10.0%)	20 (100)	
	Fast‐food chain	13 (81.3%)	3 (18.8%)	0 (0.0%)	16 (100)	
	Local fast‐food	31 (32.3%)	42 (43.8%)	23 (24.0%)	96 (100)	
	Cafe	5 (22.7%)	4 (18.2%)	13 (59.1%)	22 (100)	
	Supermarket	1 (12.5%)	4 (50.0%)	3 (37.5%)	8 (100)	

*Note:* Statistical analysis conducted using a Fisher's exact test.

### 3.7 Relationship Between Menus, Policies and Advertisements

There was no significant correlation between proportions of ‘green’ (*r*_s_ = 0.019, *n* = 18, *p* = 0.940), ‘amber’ (*r*_s_ = 0.145, *n* = 18, *p* = 0.565) and ‘red’ (*r*_s_ = −0.124, *n* = 18, *p* = 0.623) menu items and the prevalence of corresponding ‘green’, ‘amber’ and ‘red’ advertisements. Additionally, there was no significant difference in the number of advertisements surrounding schools based on the presence or absence of a *Water and Milk only Policy* (*p* = 0.877) or a *Healthy Food and Drink Policy* (*p* = 0.476).

## 4 Discussion

This investigation revealed a high prevalence of unhealthy ‘non‐core’ advertisements, particularly sugar‐sweetened beverages like Coca‐Cola, and the presence of fast‐food and convenience stores around a sample of primary schools in NZ. Non‐core advertisements were more common than core ones, and most were located near low‐ and medium‐decile schools, with the highest concentration at low‐decile schools. Additionally, the types of advertisements and food stores varied by decile. Although the study's scope was limited to 18 of the 1942 primary schools in New Zealand, the findings offer valuable insights into the food environments surrounding primary schools.

Nearly two‐thirds (61%) of schools had food and beverage advertisements within 800 m. Notably, most of the schools lacking advertisements were in rural areas, corroborating the outcomes of prior research [18]. The limited availability of food and beverage stores and advertisements in rural regions may account for this pattern. Other schools without advertisements in urban areas were of medium and high decile, situated in areas with fewer roads and lower housing density, resulting in fewer food and beverage stores and advertisements. Most advertisements were observed around Auckland schools, NZ's largest city (*n* = 375, 78.3%).

There were slightly more advertisements further away (*n* = 286, 59.7%) than close to schools (*n* = 193, 40.3%), a pattern consistent with earlier research conducted in NZ [10, 15]. This pattern could be influenced by housing density, as areas with higher residential concentrations may attract more advertisements near schools. The reasons for this pattern, however, remain unclear.

Nearly all advertisements were concentrated on food and beverage stores (97.1%, *n* = 465), particularly convenience stores (80.4%, *n* = 361), despite their representation of only 24.2% of all stores. This observation is consistent with prior research [16, 18], underscoring convenience stores as primary locations for food and beverage advertisements. Small food store owners often receive incentives, such as discounted products or signage, to promote specific items [36]. For some proprietors, these incentives and associated revenue are pivotal to business success, potentially justifying the prevalence of food and beverage advertising in these stores. Additionally, suppliers of high‐energy density products, commonly found in convenience stores, exhibit more active involvement in stocking and advertising than fruit and vegetable distributors [37]. The heightened engagement likely contributes to the increased advertising frequency on convenience stores relative to other food and beverage stores.

Most advertisements (89%, *n* = 426) featured ‘non‐core’ foods and beverages, in line with previous NZ studies [10, 14, 15, 16, 17, 18, 20]. Sugar‐sweetened options dominated the ‘non‐core’ beverages (40.1%), in agreement with findings from previous studies [10, 16, 17, 18]. The high demand for soft drinks, especially amongst children, is unsurprising, given their prevalence as the primary source of sugar in children's diets, with 45% consuming them weekly, particularly in more deprived areas and amongst Pacific and Māori populations [38]. These statistics are outdated, and it is expected that the situation has deteriorated over the past decade due to the growth of the soft drink industry [39] and rising childhood obesity rates [1]. Globally, 16 countries have implemented policy to restrict unhealthy food and beverage advertisements to children [40], in line with WHO guidelines [28]. Such policy is needed for implementation in NZ to reduce the exposure of unhealthy advertising to NZ children.

The most prominently featured brand advertised was Coca‐Cola, with 33% representation, followed by Tip‐top (*n* = 58, 12%), Monster (*n* = 34, 7%), Anchor (*n* = 32, 7%) and Streets (*n* = 29, 6%). A previous study also highlighted Coca‐Cola as the dominant brand, accounting for 17.6% of all food and beverage advertisements [16]. However, as alcoholic beverage advertisements were also included, this may have reduced the overall percentage. Given its significant advertising budget and vast resources, it is no surprise that Coca‐Cola dominates the market [39].

Out of the 430 collected advertisements, 49 (11.4%) utilised secondary marketing techniques, consistent with a recent study where promotions and discounts constituted 10.1% of all advertisements [10]. Of the 49 advertisements that employed secondary marketing techniques, the most common found was promotions/discounts, which comprised 67.3% of all marketing techniques. Promotions/discounts hold considerable sway over consumers, particularly children, who are more susceptible to persuasion and potential consumption impact [41]. Consequently, understanding the impact of secondary marketing techniques on children's food choices is crucial for formulating effective public health policies.

The NZ Advertising Standards Authority has a code regulating advertising to children and young people. Rule 1(i) prohibits occasional food and beverage advertisements from being targeted to children or in locations where children gather, such as schools [25]. Nevertheless, the current study revealed that advertisements marketed as ‘child only’ featuring the Cookie Time Monster appear near schools (*n* = 13, 2.7%), with all other advertisements surrounding schools marketed at both children and adults. These findings support previous research, in which ‘child only’ marketing accounted for 2.4% of advertisements [10]. Findings from a systematic review demonstrated that brand mascots, such as the Cookie Time Monster, wield a considerable influence on children's preferences, choices and consumption patterns, which poses a significant concern [42]. In addition, eight advertisements were found to have exaggerated portion sizes, which contravenes Rule 1 (K) of the NZ Advertising Standards Authority code. This rule requires advertised food quantities to align with appropriate portion sizes as defined by Health NZ. Hence, these advertisements present a false notion of the correct portion size to children, which could encourage them to consume more than is recommended. Inconsistency between the code and the advertisements surrounding schools suggests that the self‐regulatory nature of the code is ineffective [26]. It relies on individuals filing complaints for enforcement, making it a social rather than a governmental responsibility. Hence, there is a pressing need for increased government intervention to address limitations on occasional food and beverage advertising, especially in children's environments [43].

Studies investigating types of food and beverage stores surrounding schools in NZ have yielded mixed results. Whilst some studies suggest a higher prevalence of convenience stores compared to fast‐food stores surrounding schools [11, 12, 19], other research [13] as well as results of this study have found fast‐food stores to dominate (52.1%), over convenience stores (24.2%). Comparing studies is challenging, however, due to variations in the definitions of convenience stores across studies. Some studies include service stations within the category of convenience stores [11, 12, 19], whilst one included fruit and vegetable stores as convenience stores [13]. Additionally, differences in the location of these studies, with two in specific regions of NZ [11, 13] may have resulted in differential results.

A higher proportion of advertisements were observed within the 0–400 m category surrounding low (57.5%), followed by medium (24.4%) and high (18.1%) decile schools, trends also revealed in previous research [10, 16, 18]. Statistical testing revealed school decile to be associated with advertisement proximity. Although both low and medium‐decile schools did not differ significantly from each other, they did differ significantly from high‐decile schools in both the 0–400 m and 400–800 m categories, demonstrating a socioeconomic gradient. These findings could be attributed to the significant wealth gap in NZ, separating low and medium‐decile schools, notably from high‐decile schools [44]. Moreover, the pattern may be influenced by the geographic placement of these schools on main roads. A Canadian study found that schools with lower median income are often closer to main roads than their higher‐income counterparts, potentially explaining the observed differences in advertisement proximity, as main road areas are frequently characterised by a high prevalence of food and beverage stores and advertisements [45].

The present study found no significant differences in the amount of ‘core’ and ‘non‐core’ food and drinks advertised in schools across different decile categories. Whilst one study [14] supports these findings, others [10, 15] do not. To fully understand this trend, there is a need for further research that examines all advertisements, not just those featuring non‐core foods and beverages.

Most advertisements were found surrounding low (43.0%) and medium (43.4%) decile schools. However, an examination of Coca‐Cola advertisements relative to school decile revealed significant differences seen in the number of Coca‐Cola advertisements between low (51.3%) and medium (36.1%) decile schools. Recent research has revealed that Coca‐Cola has increased its market activities in low‐income groups of people as part of its marketing strategy due to the stagnant sales amongst people of high socioeconomic status [39]. In addition, Coca‐Cola is recognised for its marketing efforts directed at children and young adults [46]. Therefore, the increased number of Coca‐Cola advertisements around low‐decile schools in low socioeconomic neighbourhoods is unlikely to be coincidental.

The absence of a significant association between school decile and primary marketing classification is consistent with the outcomes of a recent study conducted in NZ [10]. However, the proportion of advertisements categorised as ‘child only’ was higher around low‐decile schools (46.7%) than medium and high‐decile schools (26.7%). This finding is supported by evidence suggesting lower‐income communities and children are target markets for various brands, products, and advertisers; these communities are likely subject to intentional targeting [39].

Secondary marketing classifications were weakly associated with decile, with differences observed between medium and high‐decile schools. Medium‐decile schools had a higher percentage of advertisements with secondary marketing categories (59.2%) than high‐decile schools (2%). On the other hand, medium‐decile schools had a higher percentage of advertisements without secondary marketing categories (41.6%) compared to high‐decile schools (14.9%). These results show a complex relationship between the decile level and advertisements, highlighting the need for further investigation.

Low‐decile schools had higher proportions of convenience stores (51.9%) and fast‐food chains (81.3%) compared to medium and high‐decile schools, mirroring other NZ studies [12, 13, 19, 47]. This pattern may be due to the geographic proximity of low‐decile schools to main roads, where these establishments are typically situated [45]. Additionally, the heightened demand for these stores in lower‐income neighbourhoods prompts retailers to choose these areas [29]. In contrast to earlier research, supermarkets were more prevalent around medium and high‐decile schools than low‐decile schools [29, 47]. Increased access to supermarkets has been found to be associated with healthier dietary patterns [48]. Therefore, limited access to supermarkets in low socioeconomic areas highlights inequities. Additionally, cafes are more numerous around high‐decile schools, likely driven by higher‐income households' increased spending in such establishments [49].

We found no correlation between the proportion of ‘green’, ‘amber’ and ‘red’ menu items with corresponding advertisements or between the number of advertisements surrounding schools and the presence or absence of food and drink policies within schools. This may be attributed to factors such as small sample size, government funding and support allocated to lower socioeconomic schools, and the geographical placement of schools. This lack of correlation indicates a disconnect between school policies and the surrounding food environment, suggesting that the efforts made within schools to promote healthy eating may be undermined by the prevalence of unhealthy options in the vicinity.

This study has several limitations. There was a 4‐year data gap in Google Street View, which limited coverage, and some areas, such as parks, could not be captured due to image obstruction. The 4‐year gap may have meant that neighbourhood demographics (such as SES, gentrification) may have shifted over time, thus influencing the findings. Moreover, there may have been partial obstruction affecting image comprehensiveness, and an inability to capture areas like public parks. The analysis focused solely on the main school entrance, potentially overlooking advertisements and stores at alternative entrances. Whilst categorical data improved comprehension, it sacrificed detail, and condensing categories to meet Chi‐square test assumptions may have affected result precision. Only 18 schools provided both internal food policy information and menus, which limited the opportunity to explore relationships between the external and internal school food environment. The small sample size might not fully represent NZ primary schools, and the study's narrow focus on outdoor advertisements excluded other advertising mediums. Moreover, food advertisements are temporary and subject to frequent change, and therefore our findings need to be treated with caution.

Future research with larger samples is essential, particularly to explore regional disparities across NZ, food consumed through school canteens with advertising exposure and to consider other advertising channels, such as online and TV ads, since outdoor advertisements represent only one aspect of the advertising landscape.

To protect children from unhealthy food marketing, comprehensive policy changes are necessary. Establishing zones around schools where unhealthy food outlets are limited or banned has proven effective in countries like South Korea, the United Kingdom, and the United States. In NZ, implementing a policy that prohibits new fast‐food and convenience stores from opening within an 800‐m radius (approximately a 10‐min walk) of schools could significantly reduce children's exposure to unhealthy advertising.

In conclusion, we found ‘non‐core’ food and beverage advertising, local fast‐food, fast‐food chains and convenience stores were prevalent surrounding primary schools. Regulatory approaches are needed to circumvent these occurrences. The approaches that target low and medium‐decile schools should be explored further as the non‐core food advertising was more prevalent within these lower SES areas.

### 4.1 So What?

The present study found that primary school‐aged children in NZ are potentially exposed to high levels of unhealthy food and beverage advertising and stores surrounding schools. Given the high childhood obesity rates and evidence which links adverse school food environments to children's health, implementing WHO‐recommended policies, restricting advertising to children as well as restricting new fast‐food and convenience stores near schools is recommended.

## Ethics Statement

Ethics approval was provided by Massey University (Low‐Risk approval 4000027336).

## Consent

The authors have nothing to report.

## Conflicts of Interest

The authors declare no conflicts of interest.

## Data Availability

The data that support the findings of this study are available from the corresponding author upon reasonable request.
